# Clinical evaluation and microbiota analysis in 9 dogs with antibiotic‐responsive enteropathy: A prospective comparison study

**DOI:** 10.1111/jvim.16443

**Published:** 2022-05-27

**Authors:** Enrico Bottero, Riccardo Ferriani, Elena Benvenuti, Pietro Ruggiero, Simona Astorina, Marco Giraldi, Loris Bertoldi, Giuseppe Benvenuto, Eleonora Sattin, Paola Gianella, Jan S. Suchodolski

**Affiliations:** ^1^ Endovet Group Rome Italy; ^2^ Ospedale Veterinario San Francesco Milan Italy; ^3^ Clinica Veterinaria Città di Catania Catania Italy; ^4^ MyLav LaVallonea Rho Italy; ^5^ BMR Genomics Srl Padua Italy; ^6^ Department of Veterinary Science University of Turin Grugliasco Italy; ^7^ Gastrointestinal Laboratory, Department of Small Animal Clinical Science Texas A&M University College Station Texas USA

**Keywords:** chronic diarrhea, dysbiosis, *Enterobacteriaceae*, tylosin

## Abstract

**Background:**

Antibiotic‐responsive enteropathy (ARE) is diagnosed by excluding other causes of diarrhea and when there is a short‐term response to administration of antibiotics.

**Objectives:**

To characterize the gut microbiota and clinical trend of dogs with suspected ARE and to evaluate the variation in microbiota before (T0), after 30 days (T30) of tylosin treatment, and 30 days after discontinuation of treatment (T60). A further objective was to evaluate whether changes in gut microbiota are related to relapses of diarrhea when the therapy is tapered.

**Animals:**

Study sample (group A) was composed of 15 dogs with chronic diarrhea, group B was composed of 15 healthy dogs. Group A was given tylosin for 30 days.

**Methods:**

A multicentric prospective study. Clinical Indexes, fecal score, and samples for microbiota analysis were collected at T0, T30, and T60 in group A and T0 and T30 in group B. The gut microbiota was analyzed via 16S ribosomal RNA gene. Qiime2 version 2020.2 was used to perform bioinformatic analyses, and Alpha‐ and Beta‐diversity were computed.

**Results:**

Diarrhea recurred after T30 in 9 of 14 dogs, which were classified as affected by ARE. At T0, a difference was noted in the beta‐diversity between groups (Bray Curtis metric *P* = .006). A T0‐T30 difference in alpha‐diversity was noted in group A (Shannon index *P* = .001, Faith PD *P* = .007).

**Conclusions and Clinical Importance:**

Although tylosin influences the microbiota of dogs with ARE, we failed to find any specific characteristic in the microbiota of dogs with ARE.

**Abbreviations**
ARE: antibiotic‐responsive enteropathy
BCS: body condition score
CCECAI: canine chronic enteropathy clinical activity index
FRE: food‐responsive enteropathy
IRE: immunosuppressive‐responsive enteropathy
WSAVA: World Small Animal Veterinary Association

## 1 INTRODUCTION

Chronic diarrhea is a common clinical sign in dogs with inflammatory enteropathy.^1^ On the basis of the clinical response, chronic inflammatory enteropathies are currently classified as food‐responsive enteropathy (FRE), antibiotic‐responsive enteropathy (ARE), and immunosuppressive‐responsive enteropathy (IRE).^2^, ^3^

Antibiotic‐responsive enteropathy is frequently identified in young large‐breed dogs, where certain breeds such as the German Shepherd Dog and Shar‐pei are overrepresented.^4^ Despite this, the primary underlying cause still needs to be identified.^5^ Considering that the clinical presentation is indistinguishable from other forms of chronic enteropathies, ARE is currently diagnosed by excluding other causes of chronic diarrhea, primarily FRE. Characteristics of ARE are a notable response to empiric treatment with antibiotics,^6^ followed by a relapse of the diarrhea weeks or months after the therapy has been tapered, and effective clinical response to antibiotics during each relapse of diarrhea.^4^

The term tylosin‐responsive diarrhea refers to a subgroup of AREs in which chronic diarrhea resolves using tylosin and then recurs, in a variable time period, when the drug has been discontinued.^7^ In recent years, advances in the study of the microbiota have broadened the knowledge of gastroenterology, especially as regards dogs with chronic enteropathy.^8^, ^9^

Dysbiosis is a fundamental pathophysiological element in chronic intestinal inflammation. However, it is still not entirely clear when and whether it causes intestinal inflammation, or is a consequence or both.^4^ Antibiotics show to change the composition of the gut microbiota by diminishing its richness, taxonomic diversity and contributing to dysbiosis.^10^, ^11^ Both metronidazole and tylosin are commonly used as a part of the treatment in chronic enteropathies in dogs.^10^, ^11^ Despite clinical improvements, these antibiotics can cause dramatic alterations of the microbiota after the administration period and for a long duration, even in healthy dogs.^10^, ^12^ The resilience of the microbiota means that these changes are usually temporary; however, the dynamic changes to the microbiota are unclear. Furthermore, the changes to the microbiota during chronic enteropathies and their correlation with the clinical response in enteropathic dogs are unclear.

The first aim of the study was to characterize the basal gut microbiota and the clinical trend of young adult dogs with suspected ARE and to compare it with a control group of healthy individuals matched by age and size. A further evaluation with a breed‐matching of a sub‐group of German Shepherd dogs was also performed. The second aim was to evaluate the variation in the microbiota in suspected ARE dogs before (T0), after 30 days (T30) of tylosin therapy, and 30 days after its removal (T60). In addition, differences between the microbiota of German Shepherds and other breeds were evaluated at each time‐point. The third aim was to identify whether changes in gut microbiota are associated with relapses of diarrhea when the therapy is tapered with a clinical follow up after 120 days (T120).

## 2 MATERIALS AND METHODS

### 2.1 Type of study

This is a multicentric prospective comparison study of privately owned dogs. The owners were informed of the purposes of the study and signed an informed consent form. The study received the official approval of the animal welfare committee of the University of Turin (OPBA number 1974). The treatment was part of the usual therapeutic protocol, and no invasive procedures were carried out on the dogs.

### 2.2 Animals

#### 2.2.1 Group A (dogs with chronic diarrhea)

Between February 1, 2019, and March 30, 2020, client‐owned dogs aged from 1 to 6 years old of any breed presenting with chronic diarrhea were eligible for inclusion in the study. A standard questionnaire was created a priori (Appendix) and only dogs whose questionnaire answers fulfilled the criteria were included. We defined chronic diarrhea as the presence of an abnormal fecal score (≤4), with manifestations and characteristics of both the small and large intestine, persistent or intermittent for at least 2 months in the previous 6 months. Clinical, dietary, and pharmacological histories were collected for each dog. A diagnosis of FRE was previously excluded in all dogs through at least 2 food trials with a novel monoprotic and hydrolyzed diet for at least 2 weeks. Numerous dogs were additionally fed with a low‐fat diet or hyper digestible diet. All dogs underwent extensive laboratory analyses (CBC, biochemistry, urinalysis, cTLi, folate, cobalamin, basal cortisol or ACTH stimulation test and parasitological examination of stool). All dogs had received fenbendazole treatment (Panacur forte 500 mg, MSD animal health S.r.l., Segrate, IT) of 50 mg/kg for 5 days within 2 months before inclusion. All dogs had previously undergone a histological examination classified according to the modified World Small Animal Veterinary Association (WSAVA) criteria.^13^ The dogs had also been subjected to treatment with antidiarrheals, probiotics, and immunosuppressants with various products (prednisolone at a dosage ranging from 0.5 to 1 mg/kg every 12 hours [Prednicortone 20 mg, Dechra Veterinary Products S.r.l., Turin, Italy] or azathioprine dosage 1 mg kg every day [Azatioprina 50 mg Aspen, Aspen Italia S.r.l, Verona, Italy]) and for varying periods of time. All the dogs had also been previously treated with antibiotics with temporary remission; however, no antibiotic or probiotic was used in the 45 days before inclusion in the study.

At the time of inclusion in the study, the Canine Chronic Enteropathy Clinical Activity Index (CCECAI), body condition score (BCS), age, body weight, and fecal score (point 1‐7) were recorded for each dog, which were all present with diarrhea (Table 1).

**TABLE 1:** Median age and weight, BCS, fecal score grading, and CCECAI of both groups A and B

	CCECAI (index)	BCS (1–9)	Fecal score (1–7)	Age (years)	Weight (kg)
Group A					
T0 (n. 15)	6 (5‐8)	3 (3‐5)	5 (4‐7)	2.7 (1‐6)	29.2 (17‐46)
T30 (n. 14)	1 (0‐5)	4 (3‐5)	2 (2‐6)		29.7 (18‐46)
T60 (n. 14)	4 (0‐7)	4 (3‐5)	2.4 (2‐6)	
T120 (n. 14)			3.7 (2‐7)		
Group B					
T0 (n. 15)	0	5 (4‐6)	2 (2‐3)	3 (2‐4)	29.9 (22‐40)
T30 (n. 15)	0	5 (4‐6)	2 (2‐3)		

*Note*: Group A: dogs with chronic diarrhea; group B: healthy dogs. (n.) indicates the number of subjects evaluated at that time point.

Abbreviations: BCS, body condition score; CCECAI, Canine Chronic Enteropathy Clinical Activity Index.

Exclusion criteria were: dehydration >7%, signs of systemic inflammation or other systemic diseases, endocrinopathies, hospitalization, intestinal parasites, and intestinal disorders of other etiologies (eg, mechanical obstruction from intussusception, foreign bodies or intestinal tumors), exocrine pancreatic insufficiency, and protein‐losing enteropathy.

#### 2.2.2 Group B (healthy dogs)

Client‐owned healthy dogs aged from 1 to 4 years of any breed (group B1) were included during the same period as group A.

A group of healthy German Shepherds (group B2) aged from 1 to 3 years were also included.

To determine the health status, a clinical and pharmacological history was collected and no abnormalities were detected on a clinical examination. Special focus was reserved for the presence of any sign of gastrointestinal disease. The health status was also checked again with an owner interview after 30 days.

CCECAI, Diet, BCS, age, body weight, and fecal score were recorded. Dogs were excluded from the group of healthy dogs if they were not on regular deworming therapy, showed any clinical signs or with a known disease, as well as dogs undergoing antibiotic, probiotic or prebiotic therapy in the 45 days before inclusion in the study. For dogs under an industrial maintenance diet for adult dogs of different brands, no changes in diet were recorded during the study period.

### 2.3 Therapy

In group A, tylosin (Tylan Soluble, Eli Lilly Italia S.p.a, Florence, IT) was administered at a dosage of 10 mg/kg twice a day (every 12 hours) for 30 days.^14^ All other therapies were also recorded when present. No diet was changed in any of the dogs. Any occurrence of adverse effects during therapy was recorded.

### 2.4 Collection and storage of samples

Notably, 2 to 4 g of feces was carefully collected within 10 seconds after natural defecation with a sterile cotton bud (avoiding parts in contact with the soil) and placed in a sterile tube with 1 mL of preservative medium (BEAVER Biomedical Engineering Co., containing guanidine thiocyanate, sodium chloride, and Tris‐EDTA). The sample was frozen at −80° within 5 days of collection.

GROUP A: specimens were collected by the owner, according to our instructions, at T0 (the baseline before starting the tylosin therapy), T30 (after 30 days), and T60 (30 days after the therapy was stopped).
GROUP B: specimens were collected by the owner at T0 and T30 (30 days after first collection).

### 2.5 Follow‐up

BCS, CCECAI, fecal score (points 1‐7), and general health status were recorded in the clinic at T30 for groups A and B and T60 for group A (Table 1). A clinical or telephone check‐up was also performed at T120 (2 months after T60) to assess the presence of diarrhea and any medical therapies in place.

Diarrhea was defined in dogs with a fecal score of ≥4. A dog was also considered to be affected by ARE when diarrhea relapsed without antibiotic therapy between T30 and T120.

### 2.6 Microbiota analysis

The gut microbiota was analyzed and DNA was extracted using Cador Pathogen 96 QIAcube HT Kit (Qiagen) with a lysis step modification according to Mobio PowerFecal kit (Qiagen) protocol. DNA was diluted 1:5 and 5 μL was used for the amplification. The V3‐V4 regions of the 16S ribosomal RNA gene were amplified using Illumina tailed primers Pro341F (5′‐TCGTCGGCAGCGTCAGATGTGTATAAGAGACAG‐CCTACGGGAGGCAGCA‐3′) and Pro805R (5′‐GTCTCGTGGGCTCGGAGATGTGTATAAGAGACAG‐GACTACNVGGGTATCTAATCC‐3′),^15^ using HiFi Platinum Taq (Thermo Fisher Scientific Inc) via PCR (94°C for 2 minutes, followed by 25 cycles at 94°C for 30 seconds, 55°C for 30 seconds, and 68°C for 30 seconds, and a final extension at 68°C for 7 minutes). PCR amplicons were purified by Agencourt AMPure XP Beads 0.8X (Beckman Coulter, Inc, California) and amplified following the Nextera XT Index protocol (Illumina, Inc). The purified amplicons were normalized by a SequalPrep Normalization Plate Kit (Thermo Fisher Scientific Inc) and multiplexed.

The pool was purified with 1X Magnetic Beads Agencourt XP (Beckman Coulter, Inc, California), loaded on the MiSeq System (Illumina, Inc), and sequenced using the V3 kit—300PE strategy.

### 2.7 Bioinformatic and statistical analysis

Qiime2^16^ version 2020.2 was used to perform bioinformatic analyses Raw reads were trimmed by applying Cutadapt^17^ to remove residual primer sequences and then processed with DADA2 plug‐in^18^ for denoising. Default parameters were applied to DADA2 with the exception of the truncation length option, as forward and reverse reads were truncated at 265 and 240 nucleotides, respectively. The resulting Amplicon Sequence Variant (ASV) sequences were filtered out by applying a 0.05% frequency threshold in order to discard singletons and very rare sequences. Greengeens v.13‐8 and Silva v.132 databases were used to associate the taxonomy with the remaining ASVs. Alpha‐rarefaction analysis was performed considering observed OTUs and Good's coverage metrics: 22390 reads were chosen as a rarefaction threshold for the subsequent diversity analysis. Alpha and beta diversity metrics were calculated applying the methods in Qiime2. Alpha diversity indices (Observed Otus, Shannon, Faith PD) were computed for each sample. A Kruskal‐Wallis test was used to detect significant differences across the treatments. Beta diversity analysis was carried out on all the samples using various metrics, including Bray‐Curtis, Jaccard, and UniFrac (weighted and unweighted). The resulting PCoA matrices were plotted and analyzed through the “Emperor” web tool.^19^ Statistical differences over beta‐diversity matrices were evaluated using the PERMANOVA test.

In order to perform the differential analysis, the “Phyloseq” R package^20^ was used to import Qiime2 output into the R environment. The following information was imported: ASV filtered table; phylogenetic tree with a tip for each ASV; ASV taxonomy; clinical and experimental sample information.

Differential abundances were assessed using the “DESeq2” function on the Phyloseq object.^21^ “DESeq2” first estimates the taxon‐wise dispersion by maximum likelihood estimation, and then fits the dispersion trend by combining all individual estimates. Lastly, using an empirical Bayes approach, it shrinks the taxon‐wise dispersion estimates toward the values predicted by the trend curve. Differential abundant taxa were selected from the multiple samples, in each taxonomic level (family, genus and species), with an adjusted *P*‐value <.05 as the cut‐off. Heatmaps and dot plots were generated by the “pheatmap” and “ggplot2” R packages, respectively, at every time point.

## 3 RESULTS

In group A, 15 dogs were included; 7 were German Shepherds (group A2) (3 intact females, 1 spayed female, and 3 intact males), 7 were dogs from other breeds (group A1) (one of each: Border collie, Basset hound, English cocker spaniel, Czechoslovakian wolf, Bernese Mountain dog, Akita Inu, Labrador retriever), and 1 mongrel (3 spayed females, 3 intact males, and 2 neutered males).

Each dog was previously given 1 antibiotic (8 metronidazole, 2 clavulanate and amoxicillin, 1 amoxicillin, 2 enrofloxacin, and 2 tylosin) for at least 10 days with remission of diarrhea lasting between 10 and 30 days. A second antibiotic trial was performed in 7 dogs with metronidazole and in 5 dogs with tylosin always with remission of clinical signs.

In addition, 8 dogs have been previously treated with immunomodulators (7 dogs with prednisolone alone and 1 dog with prednisolone and azathioprine).

In group B, 15 healthy dogs were included of which 8 German Shepherds (group B2) (2 intact males, 4 intact females, and 2 spayed females) and 7 dogs from other breeds (group B1) (one of each: Afghan hound, American Pitt Bull terrier, Irish setter, Czechoslovakian wolf, American Staffordshire terrier, Labrador retriever, Australian shepherd) including 2 intact males, 2 neutered males, and 3 spayed females.

Median age and weight, BCS, fecal score grading, and CCECAI of both groups were reported in Table 1. No changes were made to the diet throughout the study period. All dogs were evaluated clinically at T30 and T60, while the control was performed by telephone interview at T120.

### 3.1 T0 analysis

No significant statistical difference was found in age and weight comparing group A to group B at T0 (*P* = .09), and no difference in age was identified between healthy and diseased German Shepherd dogs (*P* = .08).

**FIGURE 1:**
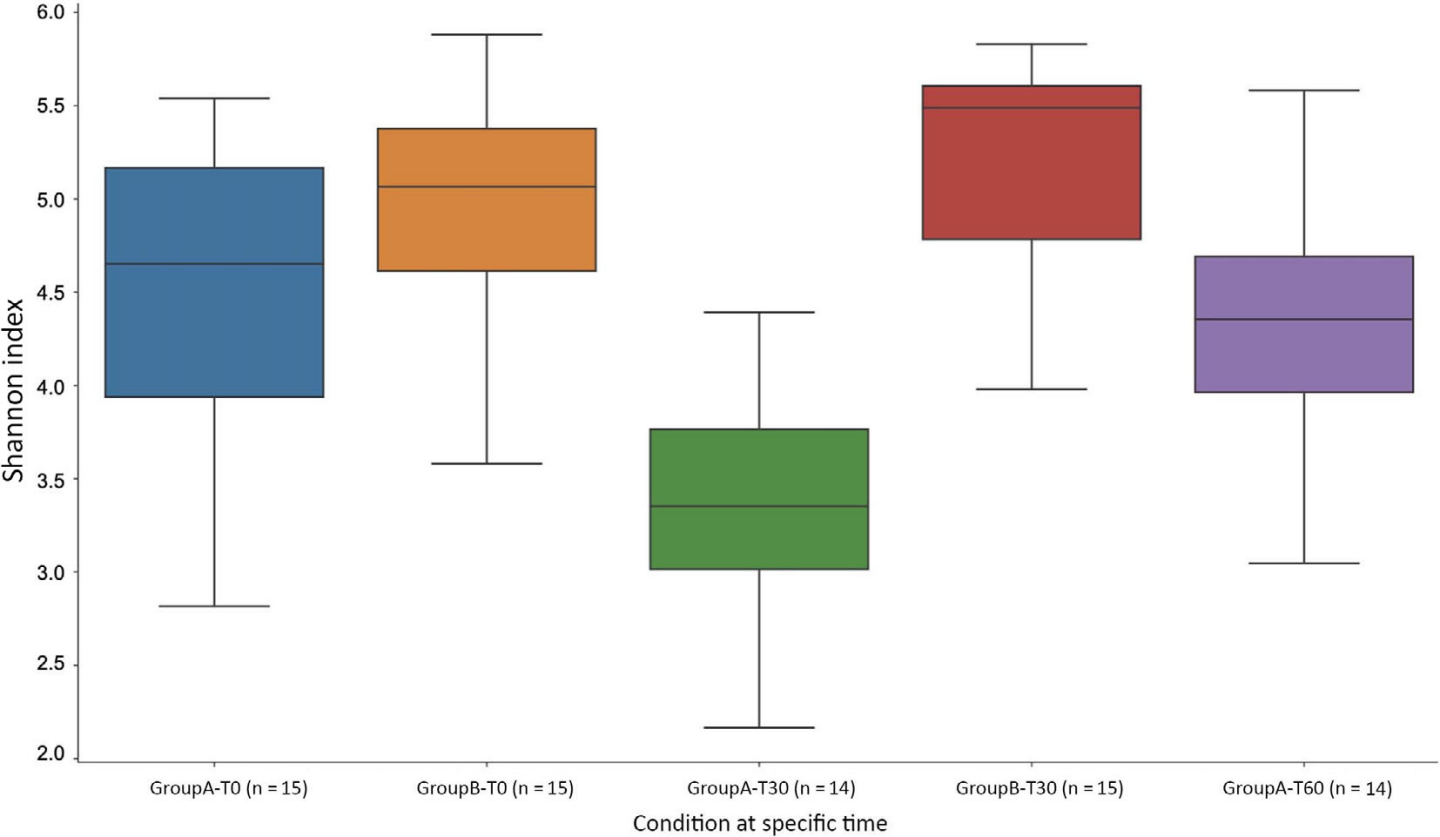
Alpha‐diversity for all dogs at T0, T30, and T60 calculated with the Shannon index. GroupA‐T0: Group A at T0 (15 dogs); GroupB‐T0: Group B al T0 (15 dogs); GoupA‐T30: Group A at T30 (14 dogs); GroupB‐T30: Group B at T30 (15 dogs); GroupA‐T60: Group A at T60 (14 dogs)

Alpha‐diversity showed no differences with the Shannon index: (*P* = .11) (Figure 1) between groups A and B at baseline (T0). Furthermore, no difference was detected between A1 and A2 dogs.

**FIGURE 2:**
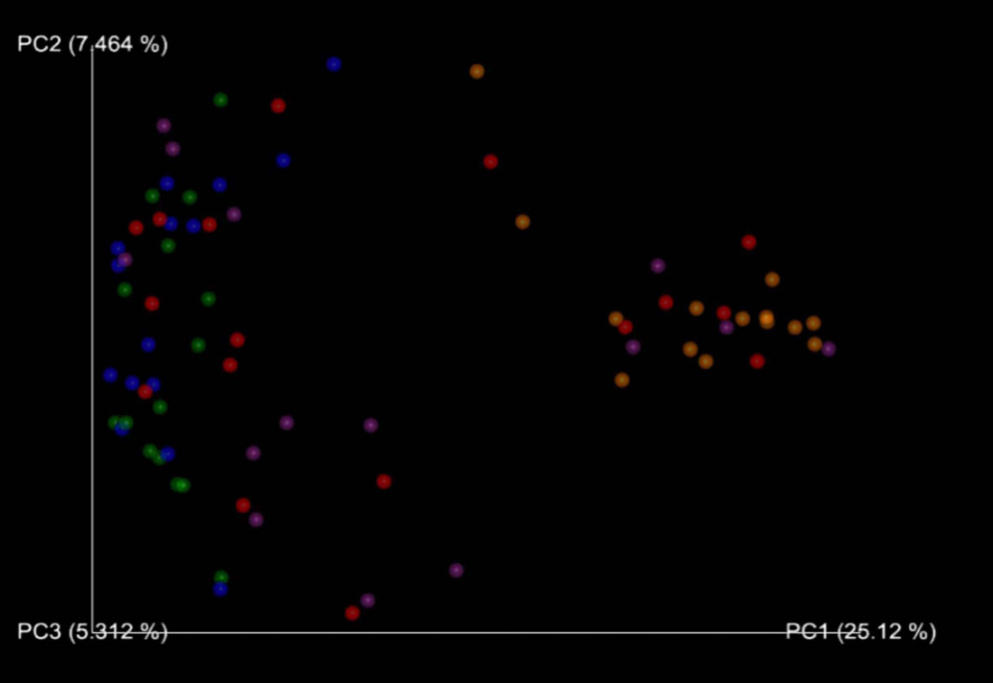
Beta‐diversity for all dogs at T0, T30, and T60 evaluated with the Bray‐Curtis method. There is a decrease in the abundance at T30 from T0. Note the graphical similarity T0‐T30 both in A‐SG1 and A‐SG2. T0_Disease_No (red spots): Group A at T0 (n. 15 dogs); T0_Heath_No (Blue spots): Group B al T0 (n. 15 dogs); T30_Disease_Yes (orange spots): Group A at T30 (n. 14 dogs); T30_Health_No (green spots): Group B at T30 (n. 15 dogs); T60_Disease_No (violet spots): Group B at T60 (n. 14 dogs)

The PERMANOVA test showed a statistical difference between group A and B when applied on the Bray‐Curtis metric (*P* = .006) (Figure 2) and unweighted UniFrac metric (*P* = .04). Heatmap analysis showed a reduced abundance in *Lactobacillaceae* in diseased dogs compared with the healthy ones.

Diseased German Shepherds were poorer in *Paraprevotella* and richer in *Lachnospiranaceae* (*Roseburia* spp and *Ruminococcus* spp) in relation to the healthy ones.

### 3.2 T30 analysis

Diarrhea resolved after 30 days in 14 dogs, and the dog in which there was no resolution was excluded from the study. No dogs from group B developed signs of gastrointestinal disease. Both dogs from Group A and Group B were in a good clinical condition.

In group B, the alpha‐diversity indices within samples (Shannon index *P* = .37, FaithPD *P =* .25) and beta‐diversity (using Bray‐Curtis metric *P* = .99, Jaccard metric *P*‐value .99, unweighted UniFrac *P =* .41) did not change significantly over time (T0 vs T30).

In group A, significant changes between T0 and T30 were observed in alpha‐diversity with a reduction in abundance (Shannon index *P =* .001, Faith PD *P =* .007) (Figure 1), but not in beta‐diversity (Bray‐Curtis metric *P =* .03, Jaccard metric *P =* .03, unweighted UniFrac metric *P* = .05) (Figure 2). Comparing T30 from group A to T0 from group B, significant changes were observed in alpha‐diversity (Shannon index *P =* .0002, Faith PD *P* = .000005) and beta‐diversity (Bray‐Curtis metric *P* = .001, Jaccard metric *P =* .001, unweighted UniFrac metric *P =* .001). No differences were detected between A1 and A2 dogs at T30.

In group A (T30), a significant increase in *Enterococcaceae* and *Enterobacteriaceae* was recorded, while *Fusobacteriaceae* were more abundant in group B (both T0 and T30). In addition, no difference was noted between German Shepherds and other breeds.

### 3.3 T60 analysis

No dog was receiving antibiotic therapy at T60. Between T30 and T60, 5 dogs from group A had a relapse of diarrhea. Of these, 1 was a German Shepherd.

Comparing T0 results vs T60 from group A, no significant alpha‐diversity was found (Shannon index *P* = .14, Faith Pd 0.81) (Figure 1) and beta‐diversity (Bray Curtis metric *P =* .08, Jaccard metric *P =* .80, unweighted UniFrac metric *P =* .91) (Figure 2).

A significant difference in alpha‐diversity with an increase in abundance was found between T30 and T60 from the same group (A) (Shannon Index *P =* .001, Faith PD *P =* .0001) and in beta‐diversity (Bray Curtis metric *P =* .001, Jaccard metric *P =* .001, unweighted UniFrac metric *P*‐value .001).

No differences in microbiota composition were found between groups A1 and A2.

No significant difference in the microbiota composition from group A was noted between dogs with and without diarrhea at T60.

### 3.4 T120 follow‐up

In the period between T60 and T120, 9 dogs from group A presented with diarrhea (Table 1).

Of these, 5 dogs presented with diarrhea at T60 and 4 developed diarrhea after T60 and before T120 (median 75 days, range, 49‐110 days). Of these, 2 were German Shepherds and 2 from other breeds.

In all dogs, treatment with tylosin was resumed and consequently the diarrhea resolved. All dogs in group B at T120 were in good condition and without diarrhea.

### 3.5 Retrospective evaluation

The 9 dogs showing relapse of diarrhea between T30 and T120 were considered to be affected by ARE.

**FIGURE 3:**
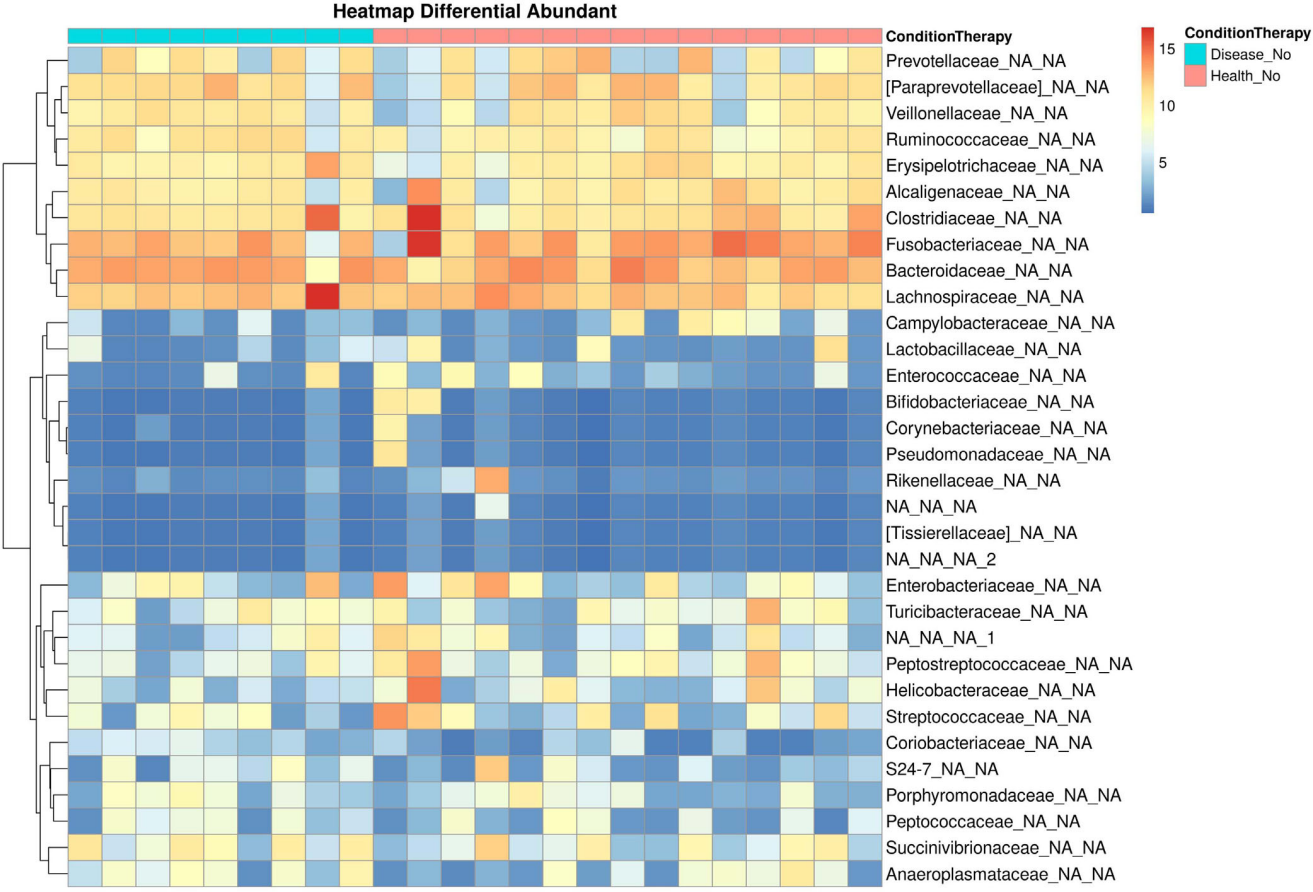
Heatmap generated at genus level from 9 ARE patients and healthy controls at T0. Every column represents a sample and every clustering represents the abundance (increasing color scale from blue to red). Antibiotic‐responsive enteropathy patients being light blue (ConditionTherapy) rectangles while healthy controls are red (ConditionTherapy) ones

Heatmaps and dot plots were generated for the evaluation of these dogs, but a specific pattern was not noted with the other 5 sick dogs at T0, T30, and T60 with the exception of *Lactobacillaceae* that overlapped in dogs with diarrhea at T120 and healthy dogs (Figure 3).

### 3.6 Drug‐related adverse events

Through an interview with the owners, the absence of adverse events related to tylosin administration in group A was verified.

### 3.7 Other therapies

During the study period, 3 of 15 dogs had subnormal (200‐400 pg/mL) and 1 of 15 had low (<200 pg/mL) levels of cobalamin and was given with cyanocobalamin (Dobetin Iniet 1000 μg/mL, Angelini S.p.a., Rome, Italy) 50 μg/kg SC every 7 days for at least 6 times.

## 4 DISCUSSION

Gastrointestinal dysfunction is the most obvious association with gut dysbiosis. The gut microbiota is altered during both acute and chronic diarrhea.^22^, ^23^ The dysbiotic alterations of the microbiota are related to intestinal inflammation and, although there are no typical dysbiotic patterns related to the different enteropathies, some specific characteristics can be found, such as the reduced abundance that occurs in enteropathic dogs compared to healthy dogs.^8^

Antibiotic therapy is often used in acute and chronic gastrointestinal diseases,^24^ despite the widely known negative dysbiotic effect.^10^, ^11^, ^25^, ^26^, ^27^

The accurate classification of subjects with ARE can be challenging and therefore, to be as objective as possible we used a standardized questionnaire (Appendix) to include subjects with clinical features compatible with the disease. We are aware of the limitations of this approach as the clinical behavior is not pathognomonic and the answers were subjective and reflected the owner's opinion.

The true prevalence of ARE is currently unknown as many antibiotics with a range of dosages and treatment lengths are in the literature. Despite this, our personal experience is that about 5% of dogs with chronic diarrhea are affected by ARE.

The first objective of our study was thus to characterize the microbiota of dogs with suspected ARE by comparing it to the microbiota of healthy dogs with similar signalment. In accordance with previous studies,^8^, ^12^ we found no differences in the evaluation (alpha‐diversity) of the microbiota at T0 between the groups of sick and healthy dogs (Figure 1).

Despite this, we found a significant difference regarding the decreased richness in *Lactobacillaceae* in dogs with diarrhea compared with healthy dogs at T0. Dogs with diarrhea at T120 classified as ARE had levels of *Lactobabillaceae* similar to healthy dogs at T0. The reason of this discrepancy in group A is not clear.

It is also possible that the whole sample of diseased dogs showed different clusters of beta‐diversity, and we did not identify a typical display that could be used to identify dogs with ARE or even with chronic diarrhea. Our dogs with chronic diarrhea did not show a repeatable metagenomic pattern.^28^ This supports the results of a previous study that found no typical or repeatable dysbiotic patterns.^4^ In fact, to date, there are no typical and repeatable dysbiotic patterns among different enteropathy phenotypes when using sequencing approaches. After 30 days, all dogs showed a remission of diarrhea.

When comparing the microbiota analysis at T30 with the T0 in dogs with the resolution of diarrhea, a significant change in alpha‐diversity was noted, although not in beta‐diversity. This is not unexpected when using an antibiotic, and this finding is consistent with other studies, albeit performed on healthy animals^10^, ^12^ or in vitro.^29^

Indeed, as already hypothesized,^7^ the reduction in microbiota induces a down regulation of an aberrant host response directed against microbial antigens. It is thus important to emphasize that the use of tylosin should be reserved for dogs with a negative response to multiple food trials and also to immunosuppressive therapy.

In our study, there was also a significant increase in *Enterococcaceae* and *Enterobacteriaceae*, while *Fusobacteriaceae* were more abundant in the group of healthy dogs.^30^ *Enterobacteriaceae* are Gram‐facultative aerobic bacteria that can exacerbate inflammation.^31^

In contrast, *Fusobacteria* are more representative of healthy microbiome in dogs and their decrement is associated with chronic enteropathies.^31^ The inconsistency of this result with a clear clinical improvement still needs clarification and further studies.

The administration of tylosin decreases *Clostridium* species in healthy dogs^10^ and specifically *Clostridium hiranonis*.^12^ This strain is considered beneficial and its decrease is related to dysbiosis and acid bile metabolism.^32^ One limitation of our study was the lack of the direct detection via PCR of *Clostridium hiranonis*, as it would have been interesting to assess the dynamic changes in *Clostridium hiranonis* in dogs with ARE.

Moreover, even bacteria considered as eubiotic, such as *Faecalibacterium prausnitzii*, are completely absent in sick dogs in clinical remission. The crucial question that remains unanswered is whether it is the reduction in some bacterial species or the increase in others that play a positive role in reducing inflammation or whether the total quantity of bacteria regardless of their type is an important pathophysiological factor.

It is also worth noting that a stronger or different action of tylosin can occur in the small intestine rather than the colon where more microorganisms and different species are present and accompanied by a different mucus layer.^4^, ^33^, ^34^, ^35^

The potential anti‐inflammatory effect of tylosin is in our opinion less likely because dogs with ARE generally do not respond well to steroid therapy and also in our series dogs treated with immunomodulation therapies not improved.

In our case series, 30 days after the suspension (T60) of antibiotics no significant difference was found in alpha‐diversity and beta‐diversity within samples when comparing T0 to T60. This is interesting because in previous studies on healthy dogs, the administration of tylosin even for less than 30 days required a longer time to restore the original microbiota setting. For instance, in one study^10^ after 28 days, only 2 out of 5 healthy dogs treated with tylosin exhibited complete microbiota resilience, while in another study^12^ resiliency was not uniformly achieved 2 months after discontinuation of tylosin.

On the basis of the recurrence of diarrhea after the discontinuation of tylosin, 9 cases of ARE were identified.

The first consideration is the risk of a false diagnosis of ARE. In fact, in 5 other dogs, the hypothesis of ARE was not confirmed by the clinical trend over the following 4 months. It clearly cannot be excluded that some cases of ARE have longer interval times in relapses than 3 months. However, the most likely probability is that these 5 dogs had other causes of enteropathy in which the dysbiosis responded to tylosin without recurring, or in which the pathological process resolved autonomously. Unfortunately, even in the 9 dogs with ARE, the evaluation of the microbiota did not enable us to identify a comparable metagenomic phenotype.

## CONFLICT OF INTEREST DECLARATION

Authors declare no conflict of interest.

## OFF‐LABEL ANTIMICROBIAL DECLARATION

Tylosin was used off‐label on the basis of the therapeutic needs of the animals and in the absence of authorized treatment and licensed alternatives.

## INSTITUTIONAL ANIMAL CARE AND USE COMMITTEE (IACUC) OR OTHER APPROVAL DECLARATION

Approved by the animal welfare committee (Commissione Etica e di Benessere Animale del Dipartimento di Scienze Veterinarie) of the University of Turin (OPBA number 1974).

## HUMAN ETHICS APPROVAL DECLARATION

Authors declare human ethics approval was not needed for this study.

## Appendix

Standard questionnaire used to survey owners and include dogs in the study:

1. What is the dog's main symptom?
2. Which therapy has shown the best clinical outcome so far?
3. Did the diarrhea relapse within 6 weeks of antibiotic discontinuation?

Dogs were included in the study only if their answer to the first question was “diarrhea,” the answer to the second question related to “antibiotic” therapy, and the answer to the third question was “yes.”
